# Validation of Three-Dimensional Magnetic Resonance Imaging-Based Volumetric Quantification for Fatty Infiltration in a Rabbit Model of Chronic Rotator Cuff Tears

**DOI:** 10.3390/diagnostics16050705

**Published:** 2026-02-27

**Authors:** Jieun Kwon, Hyeon Jang Jeong, Sheng-Chen Han, Joo Han Oh

**Affiliations:** 1 Department of Orthopaedic Surgery, Ewha Womans University College of Medicine, Ewha Womans University Mokdong Hospital, Seoul 07985, Republic of Korea; toast8315@gmail.com; 2 Department of Orthopaedic Surgery, Seoul National University College of Medicine, Seoul National University Bundang Hospital, Seongnam-si 13620, Republic of Korea; feroz9@gmail.com; 3 Center for Rehabilitation Medicine, Department of Orthopedics, Zhejiang Provincial People’s Hospital (Affiliated People’s Hospital), Hangzhou Medical College, Hangzhou 310014, China; shengchen111@hotmail.com

**Keywords:** rotator cuff tear, fatty infiltration, quantitative measurement method, rabbit model

## Abstract

**Backgrounds/Objectives:** Fatty infiltration (FI) of rotator cuff (RC) muscles is a critical prognostic factor after surgical repair. While the Goutallier–Fuchs grading system is widely used, its reproducibility is often debated. This study aimed to validate a previously reported three-dimensional (3D) magnetic resonance imaging (MRI)-based volumetric quantification method by comparing it with histologic findings in a chronic rotator cuff tear (RCT) rabbit model. **Methods:** Eighteen shoulders from nine rabbits were randomly assigned to three groups (*n* = 6 each): repair (A), chronic tear (B), and control (C). In groups A and B, a chronic RCT model was established by detaching the supraspinatus tendon, with group A receiving repair after six weeks. At 12 weeks after repair, 7.0T MRI was performed, and volumetric quantification of intra-muscular fat was performed using semi-automated 3D Slicer software. Histologic fat proportion was measured via Oil Red O staining and ImageJ analysis. **Results:** The muscle weight and MRI-based muscle volume were significantly lower in group B than group C (*p* < 0.05). The radiologically measured fat proportion was significantly higher in groups A (1.8 ± 0.8) and B (2.8 ± 0.7) compared to group C (0.5 ± 0.4, *p* < 0.001). Histologic analysis showed a corresponding pattern (3.0 ± 1.2%, 5.2 ± 1.0%, 1.7 ± 1.0% for groups A, B, and C, respectively; *p* < 0.001). A strong positive correlation was identified between the radiologic and histologic measurements of FI (r = 0.784, *p* < 0.001). **Conclusions:** Direct histologic comparison validates the reliability of 3D MRI-based volumetric quantification for evaluating FI of the RC muscle in a chronic RCT rabbit model. This objective approach may address the inherent limitations of the conventional qualitative grading system.

## 1. Introduction

Rotator cuff tears (RCTs) are a common pathologic condition that can produce shoulder pain and dysfunction [1,2]. The loss of the tendon attachment from its insertion and the subsequent unloading of tensile forces lead to a reduction in muscle mass and fatty accumulation within rotator cuff (RC) muscles [3,4]. This appearance after chronic RCTs has been described as “fatty infiltration (FI)” or “fatty degeneration” [5]. Progressed FI of RC muscles is a significant predictor for healing failure following rotator cuff repair (RCR) [6,7,8,9]. Therefore, an accurate preoperative assessment of FI is essential for surgical planning and managing patient expectations.

The Goutallier–Fuchs grading system [8,10], which is based on the percentage of fat change within the affected muscles assessed using computed tomography (CT) or magnetic resonance imaging (MRI), has been widely used to evaluate the FI of RC muscles. However, as the Goutallier–Fuchs grading system depends on the subjective visual interpretation, its reliability is questioned [11,12,13]. Additionally, information distortion may occur because conventional methods interpret the three-dimensional (3D) structure of RC muscles and fat tissues using a single sagittal-oblique two-dimensional (2D) image [12]. In our previous study [14], the authors directly assessed 3D volumetric data of RC muscles and fat tissues to overcome the limitations of the Goutallier–Fuchs grading system. In that study, we evaluated a new quantitative measurement of FI based on 3D-reconstructed volumetric data obtained from MRI of non-pathologic human shoulders using the open-source software, 3D Slicer (version 4.6.2; Slicer Community, Boston, MA, USA; http://www.slicer.org accessed on 28 March 2022). This method demonstrated excellent consistency; however, we could not measure the actual volume of RC muscles and fat tissues in humans for ethical reasons. Therefore, a further animal study was required to validate this method by comparing MRI-based 3D volumetric data with the actual muscle weight and histologic findings.

The purpose of this study was to validate the quantitative measurement of FI by comparing MRI-based volumetric data and histologic fat proportion in a rabbit model of chronic RCT. The hypothesis was that the degree of FI measured using quantitative radiologic evaluation would show a significant correlation with the histologic fat proportion of the RC muscles.

## 2. Methods

All animal care and experimental procedures in this study were conducted at the senior author’s institute and were approved by the Institutional Animal Care and Use Committee (IACUC) (BA-2112-334-002-08).

### 2.1. Allocation of Rabbits

**Figure 1:**
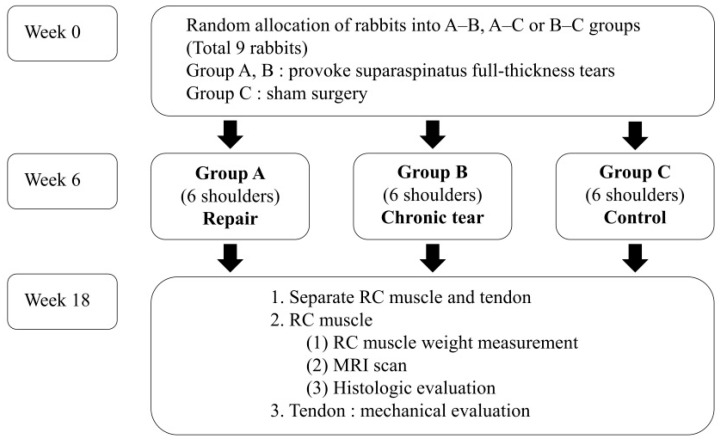
Flowchart of experimental study design. RC, rotator cuff; MRI, magnetic resonance imaging.

Since no previous study has analyzed the quantitative measurement of FI in the RC muscle using MRI in a rabbit model of chronic tears, the required sample size was calculated based on the results of previous studies comparing FI between chronic RCT and control groups histologically in a rabbit model [15,16,17]. In previous studies, the chronic RCT and control groups showed moderate or severe fatty infiltration in 100% and 12.5% of cases, respectively. According to this data, we calculated a sample size of 5 shoulders per group to detect a significant difference in the fat-to-muscle proportion (α-error = 0.05, power = 0.8). Considering a dropout rate of 20%, eighteen shoulders from nine New Zealand White male rabbits (weighing approximately 3.5–4.0 kg) were randomly allocated into three groups (6 shoulders per group): group A (repair), group B (chronic tear), group C (control) (Figure 1).

### 2.2. Surgical Procedures

Under general anesthesia, a lateral skin incision was made between the acromion process and the greater tuberosity. To create a chronic tear model in group A and B, the supraspinatus tendon was severed at the insertion site with a scalpel blade. The torn tendon was then wrapped with a 10 mm long silicon Penrose drain (8 mm in outer diameter, Yushin Corp, Seoul, Korea) to prevent adhesion to the surrounding soft tissues [16,17,18]. In group C, only a skin incision was made at this stage.

**Figure 2:**
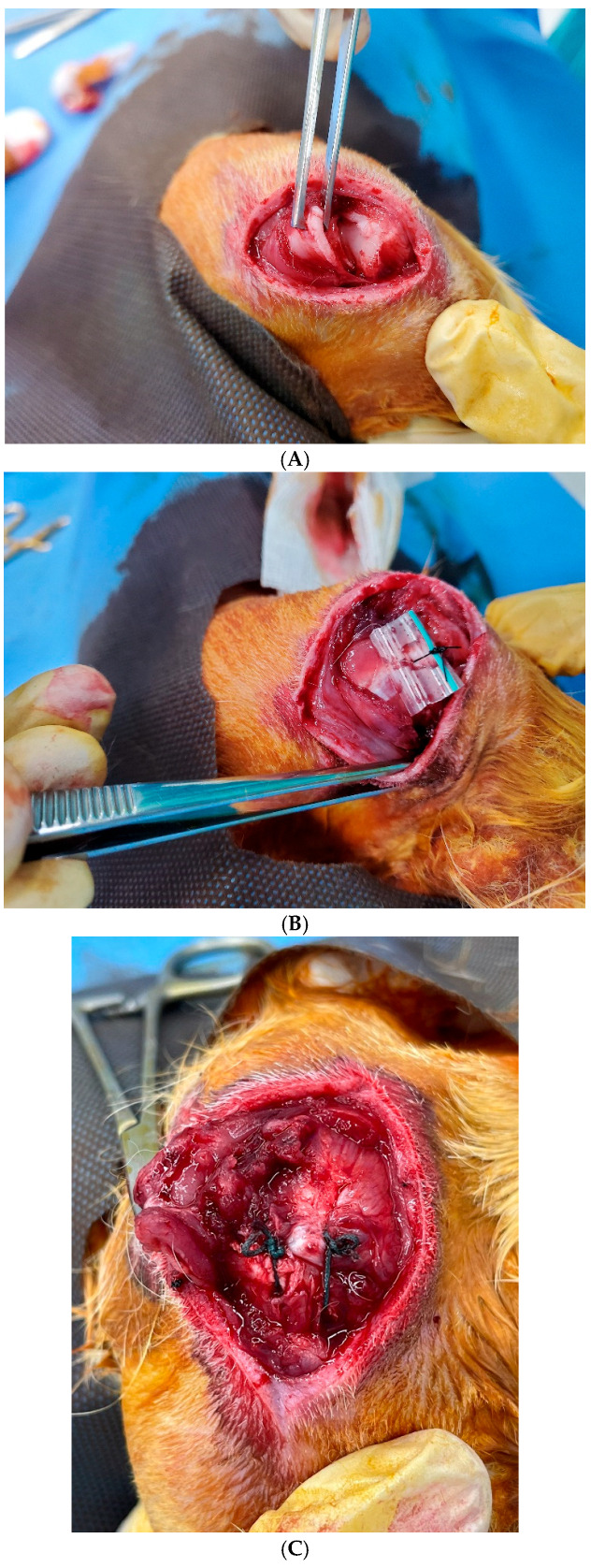
Surgical procedures for establishing a chronic cuff tear model. (**A**) Supraspinatus tendon was severed at the insertion site in groups A and B. (**B**) The torn tendon was wrapped with a Penrose drain to prevent spontaneous healing and adhesion. (**C**) Six weeks after the initial procedure, the torn tendon was repaired in group A using a transosseous technique.

Six weeks after the initial procedure, the torn supraspinatus tendon was repaired in group A. Specifically, the Penrose drain was removed, and the greater tuberosity was prepared with a scalpel. Two bone tunnels were created, and suture material (2-0 Ticron, Tyco, Waltham, MA, USA) was passed through the tunnels and tied to reattach the tendon to its footprint. In group B, the Penrose was removed without repair, and sham surgery was performed in group C (Figure 2). The wounds were closed in layers. An intramuscular antibiotic (cefazolin 30 mg/kg) was injected immediately after the operation and every 24 h for 3 days thereafter to prevent perioperative infection.

Postoperatively, the shoulders were not immobilized, allowing for unrestricted activity within the cages. Rabbits were housed individually in a temperature-controlled environment and provided with free access to food and water.

### 2.3. Measurement of Rotator Cuff Muscle Weight

**Figure 3:**
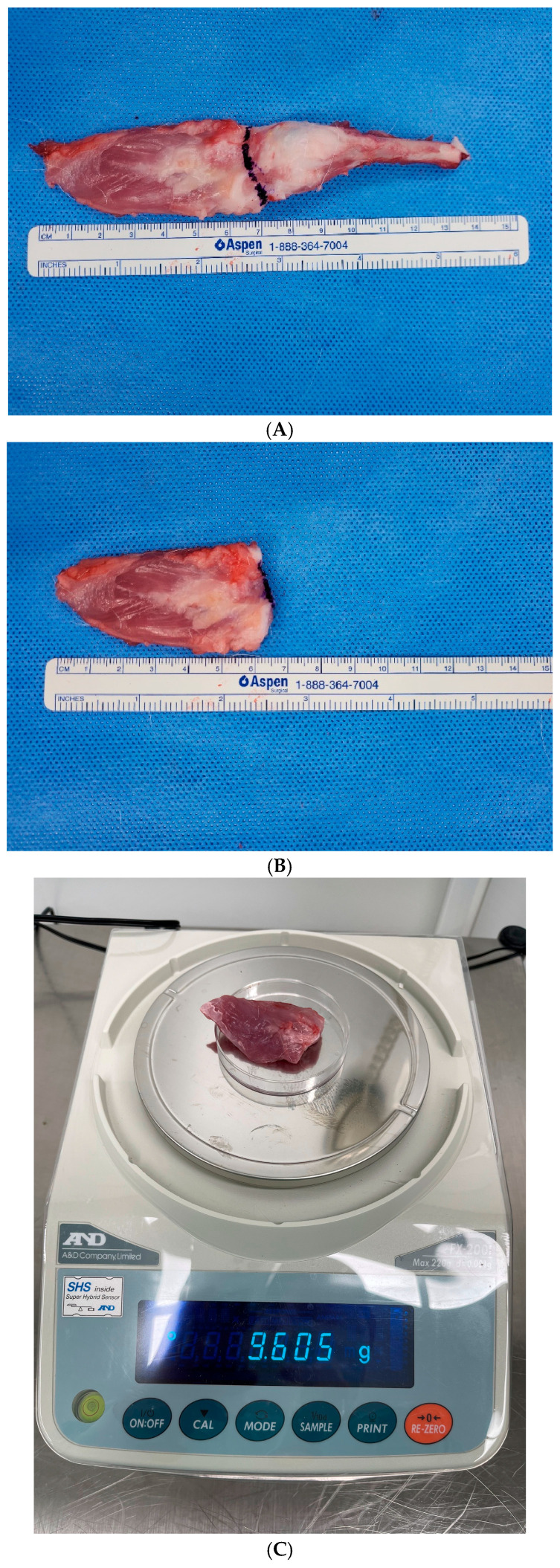
Measurement of rotator cuff muscle weight. (**A**) Harvesting of the entire supraspinatus muscle–tendon unit. (**B**). Separation of the supraspinatus muscle from the tendon at the musculotendinous junction. (**C**) Measurement of the isolated supraspinatus muscle weight using a digital scale.

Twelve weeks after the second procedure (repair for group A, non-repair for group B, and sham surgery for group C), all rabbits were fully anesthetized and euthanized with a cardiac potassium chloride injection (2 mmol/kg). The entire supraspinatus muscle and tendon were harvested from each shoulder. To ensure measurement accuracy, all non-muscular soft tissues and adherent fat were meticulously removed. The supraspinatus muscle was then separated from the tendon by a transverse section at the musculotendinous junction. The weight of the isolated supraspinatus muscle was measured to the nearest 0.001 g using an electronic precision balance (FX-200i; A&D Company, Ltd., Tokyo, Japan) (Figure 3).

### 2.4. Radiologic Evaluation

Imaging of the specimens was performed using a high-field (7.0 Tesla, 24 cm horizontal bore magnet) small-animal MR scanner (MR SOLUTIONS Ltd., Guildford, UK) within one hour of collection. A quadrature birdcage coil (inner diameter = 65 mm) was used for excitation. T2-weighted 2 mm axial slices (thickness, 2 mm) were acquired with a fast spin echo sequence (field of view [FOV], 60 mm × 40 mm; TE/TR, 45 ms/8000 ms; matrix, 256 × 238; number of excitations [NEX], 4). The total acquisition time was approximately 18 min per subject. T1-weighted axial slices (thickness, 2 mm) were also acquired (FOV, 60 mm × 40 mm; TE/TR, 14 ms/500 ms; matrix, 256 × 238; NEX, 2). The total acquisition time was approximately 22 min per subject.

The segmentation protocol was performed based on the methodology established in our previous study [14]. 3D Slicer version 4.6.2 was employed for 3D reconstruction and volumetric analysis of the muscle fat tissues. To determine the optimal threshold for tissue differentiation, the signal intensity was analyzed using the data probe function in Slicer. We measured the signal intensities at the cursor position for the muscle and fat tissues across the specimens. A total of 90 points (5 points per specimen) were sampled to provide a representative distribution of signal values. The sampled signal intensities for muscle tissue ranged from 1002 to 3448, while fat tissue intensities ranged from 3600 to 9652. Based on the receiver operating characteristic (ROC) coordinates, the statistical cutoff value was identified within the range of 3448 and 3600, yielding 100% sensitivity and 100% specificity. Considering a potential “gray zone” due to measurement errors at the tissue interface, the authors established signal intensity range of 1000–3500 for muscle tissues and 3600 or higher for fat tissues.

**Figure 4:**
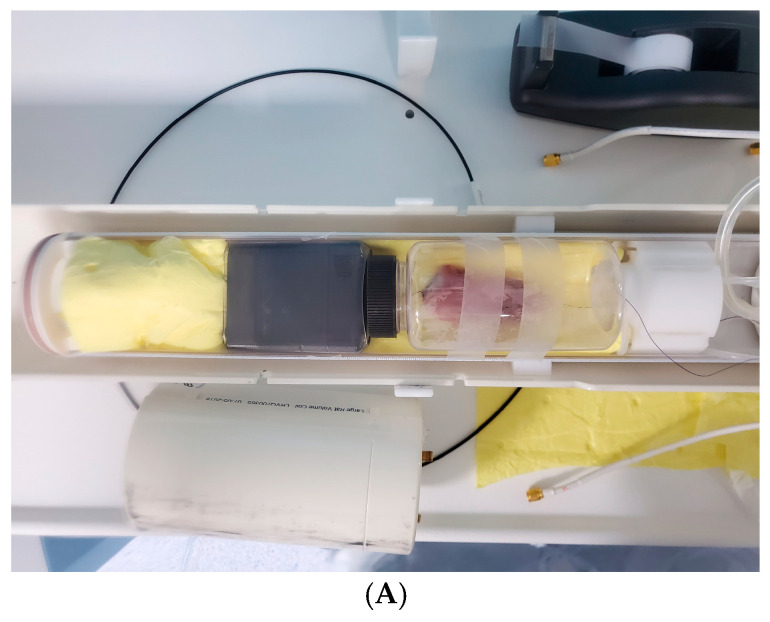

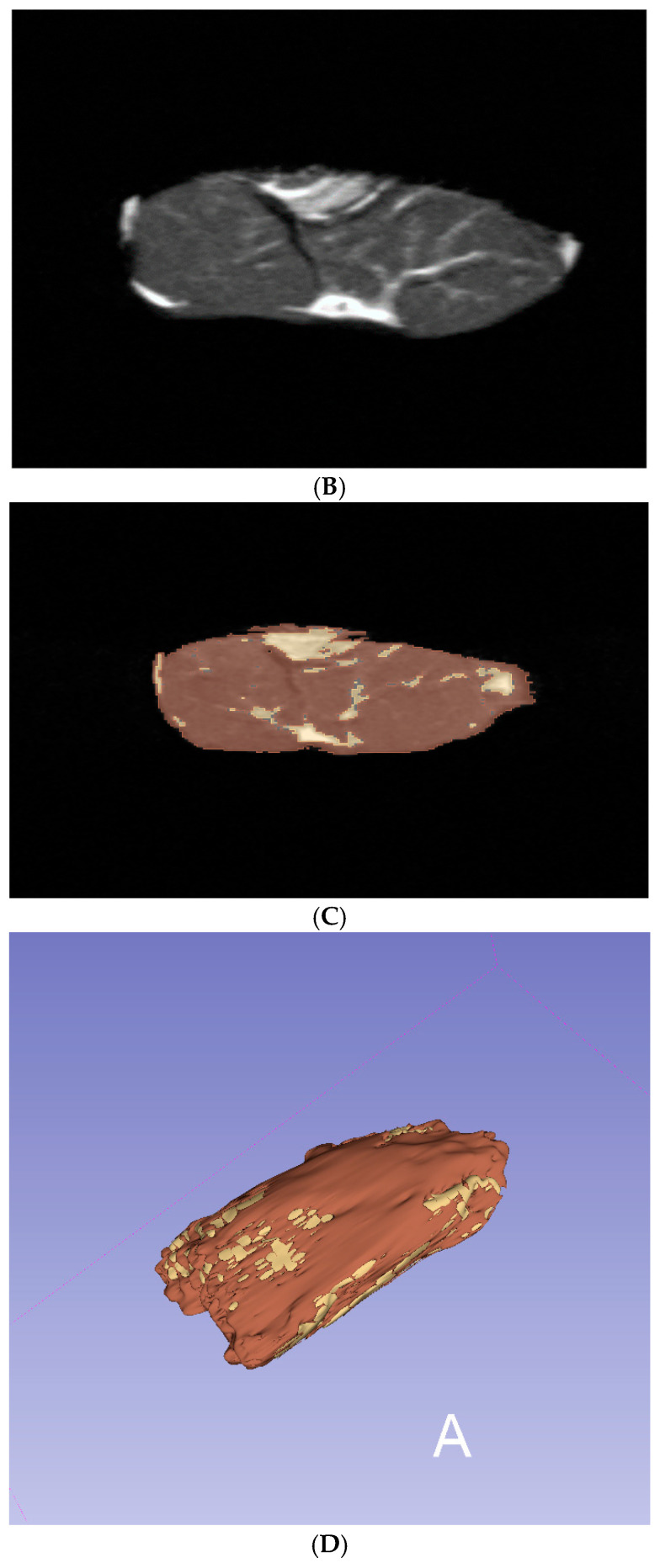
Radiologic evaluation and 3D volumetric analysis workflow. (**A**) A quadrature birdcage coil used for signal excitation and reception. (**B**) Representative raw (unprocessed) axial T1-weighted magnetic resonance image of the supraspinatus muscle. (**C**) Semi-automated segmentation of the supraspinatus muscle and fat tissues using the threshold pain function. (**D**) Three-dimensional (3D) reconstruction of muscle (brown) and fat (yellow) volume using the model maker function within the surface models module of 3D Slicer.

Segmentation was conducted using the editor module with the threshold paint function. The preset range for muscle was applied to automatically paint the supraspinatus muscle area within its anatomical contour. Subsequently, the fat tissue was segmented by applying the threshold for intensities of 3600 and above. Finally, 3D models of the muscle and fat were generated using the model maker function in the surface models module. The volumetric data for each tissue type were automatically calculated using the label statistics function within the quantification module (Figure 4).

### 2.5. Histologic Evaluation

The supraspinatus muscle was mounted in optimal cutting temperature (OCT) compound on filter paper and frozen in liquid nitrogen. The specimens were then sectioned to a 5 μm thickness using a Leica CM 1900 cryostat (Leica Microsystems Inc., Buffalo Grove, IL, USA). The sections were mounted on glass slides and stained with Oil Red O to visualize lipid deposits. The staining procedure involved incubating the sections in Oil Red O solution (ORK-1-IFU; ScyTek Laboratories Inc., Logan, UT, USA) for 10 min, followed by a thorough wash in distilled water. The sections were then counterstained with hematoxylin to delineate the nuclei. High-resolution images of the stained sections were captured at ×200 magnification using an Eclipse Ci-L microscope (Nikon, Tokyo, Japan) to ensure adequate resolution for the identification of interstitial lipid deposits.

For each slide, 10 sections were randomly scanned to provide a representative assessment of the tissue. To ensure a strictly blinded process, the randomization and coding of the slides were performed by a separate researcher who was not involved in the subsequent image analysis. All images were analyzed by a single examiner in a randomized and blinded fashion. To establish intraobserver reliability, the examiner reviewed each of the 10 scanned sections twice at two difference time points, with a minimum interval of four weeks between assessments.

**Figure 5:**
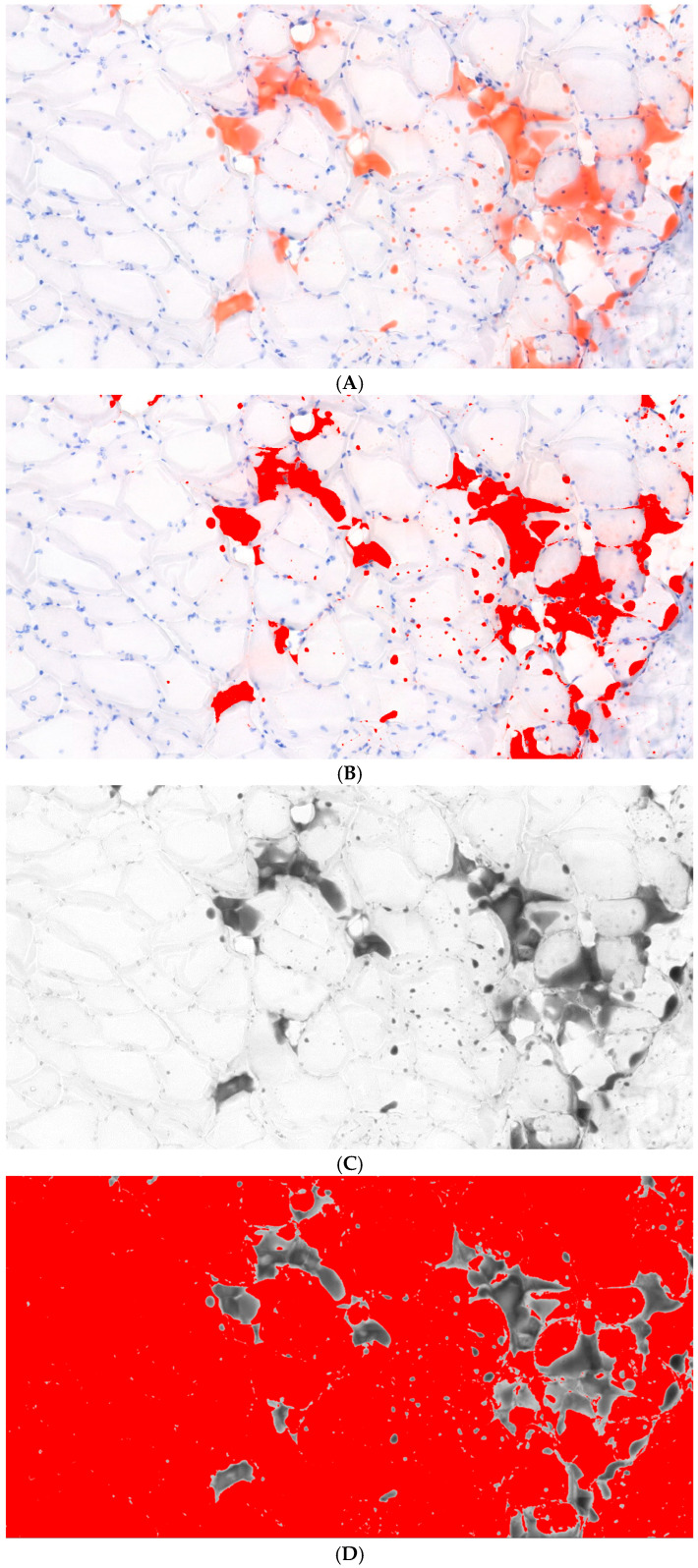
Quantitative histological analysis of fatty infiltration and muscle proportion. (**A**) Representative histological section showing hematoxylin and eosin (H&E) and Oil Red O staining. Images were captured using an Eclipse Ci-L microscope (Nikon, Tokyo, Japan) equipped with a digital camera and acquisition software (DS-U3 and NIS Elements BR version 5.2; Nikon, Tokyo, Japan) at an original magnification of ×200. (**B**) Quantification of fat area using the color threshold function in the HSB (Hue, Saturation, Brightness) color space to isolate Oil Red O-stained lipid droplets. (**C**) Selection of the green channels from the RGB image to maximize contrast for muscle tissue. (**D**) Quantification of the muscle fiber area using the threshold function applied to the green channel.

Quantitative analysis of FI and muscle proportion was performed using ImageJ software (version 1.53; National Institutes of Health, Bethesda, MD, USA) (Figure 5) [19]. For FI assessment, the color threshold function in the HSB (Hue, Saturation, Brightness) color space was utilized. For muscle quantification, the RGB channel was decomposed into individual channels, and the green channel was selected to maximize the contrast of eosin-stained muscle fibers. The average of these repeated measurement from the 10 sections was used for the final quantitative analysis. Intraobserver reliability was excellent with an interclass correlation coefficient of 0.94 (*p* < 0.001).

### 2.6. Mechanical Evaluation

**Figure 6:**
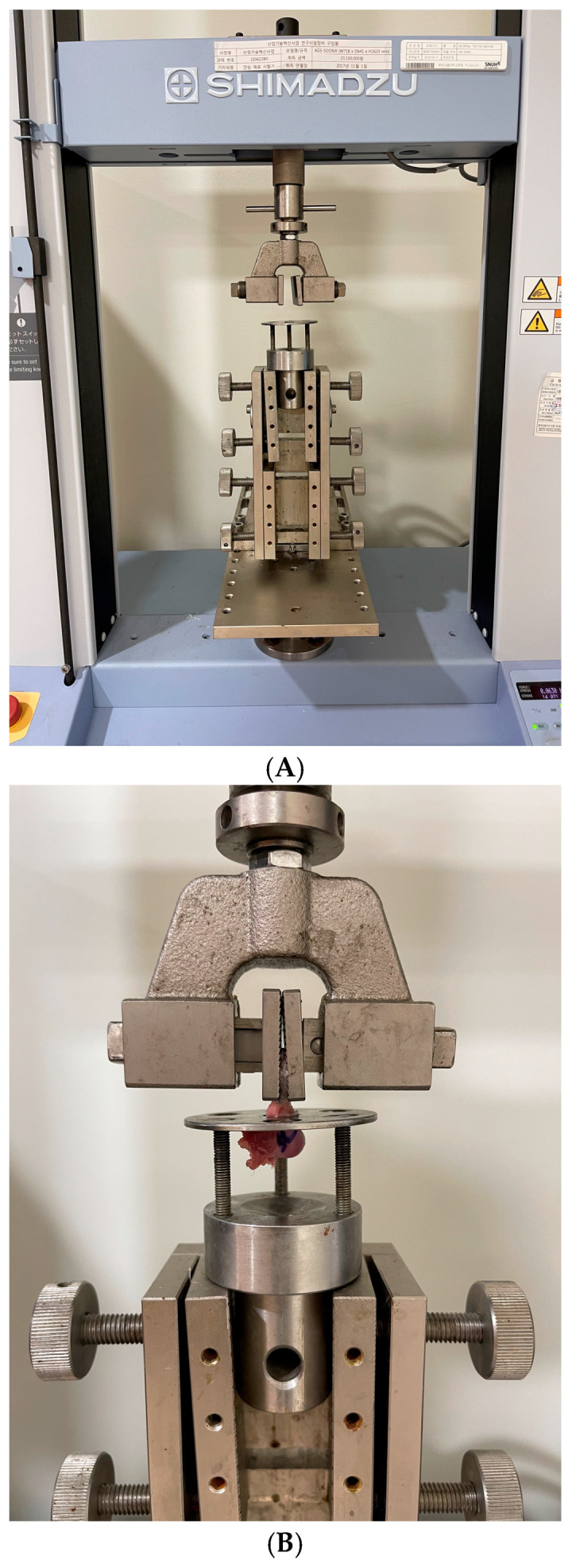
Experimental setup for biomechanical evaluation. (**A**) The universal testing machine used for tensile strength measurement. (**B**) A custom fixture-clamping system developed to ensure secure and anatomical fixation of the tendon–bone unit.

The failure mode and ultimate load-to-failure were evaluated at a crosshead speed of 1 mm/s. Before the final testing, a preload of 5 N was applied, followed by five consecutive preconditioning cycles (5–50 N at a loading rate of 15 N/s) using a universal testing machine (AGS-X; Shimadzu, Kyoto, Japan). The supraspinatus tendon along with the humeral head was firmly fixed to the system in its anatomic orientation to allow axial tensile loading. The tendon was then loaded until it pulled apart from the humeral head or ruptured at its midsubstance. Data were automatically collected on a personal computer-based data acquisition system (Figure 6).

### 2.7. Statistical Analysis

Statistical analyses were performed using SPSS software (version 20.0; IBM Corp., Armonk, NY, USA), and *p*-value < 0.05 was considered statistically significant. One-way analysis of variance (ANOVA), followed by Bonferroni post hoc tests, was used to evaluate the supraspinatus muscle weight, as well as radiologic and histologic measurements between the groups. The Kruskal–Wallis test, followed by Mann–Whitney U post hoc test with Bonferroni correction, was used to compare the results of the mechanical evaluations between the groups. The intraobserver reliability for histologic quantitative measurements was assessed using the intraclass correlation coefficient (ICC). Spearman’s correlation analysis was performed to determine the correlation between the radiologic and histologic quantitative measurements. A *p*-value < 0.05 was considered statistically significant.

## 3. Results

### 3.1. Rotator Cuff Muscle Weight Measurement

The average body weight of the rabbits was 4.0 ± 0.3 kg and the mean weight of the supraspinatus muscle was 9.4 ± 1.3 g across all subjects. The mean supraspinatus muscle weights and supraspinatus muscle-to-body weight proportion were significantly different among the three groups (all *p* < 0.05; Table 1). In the post hoc analysis, group B showed a significantly lower muscle-to-body weight proportion compared with group C (*p* = 0.002).

### 3.2. Radiologic Evaluation

The results of the quantitative measurement of supraspinatus muscle and fat volume using 3D-reconstructed volumetric MRI data are summarized in Table 2. Due to magnetic susceptibility artifacts, radiologic evaluation could not be performed on one specimen each from groups A and B. However, as the sample size calculation estimated that five specimens per group were needed to detect statistical differences, this requirement was still satisfied.

Group C exhibited the highest mean muscle volume among the three groups (*p* = 0.009). Post hoc analysis revealed that group C had significantly greater muscle volume than both groups A (*p* = 0.049) and B (*p* = 0.011). In contrast, the fat-to-muscle proportion (FTMP) was highest in group B (*p* < 0.001). This value was significantly higher compared with both group A (*p* = 0.015) and group C (*p* < 0.001). Notably, group A also demonstrated a significantly increased FTMP compared with group C (*p* = 0.015), suggesting that FI persists to some extent even after surgical repair.

### 3.3. Histologic Evaluation

The mean fat proportion increased in the order: group C, A, and B. A statistically significant difference was found among the three groups (*p* < 0.001). Post hoc analysis of the histologic data revealed that the fat proportion in group B was significantly higher than in group C (*p* = 0.001). However, no statistically significant differences were observed between group A and B (*p* = 0.053), or between A and C (*p* = 0.101) (Table 3).

### 3.4. Correlation Analysis

**Figure 7:**
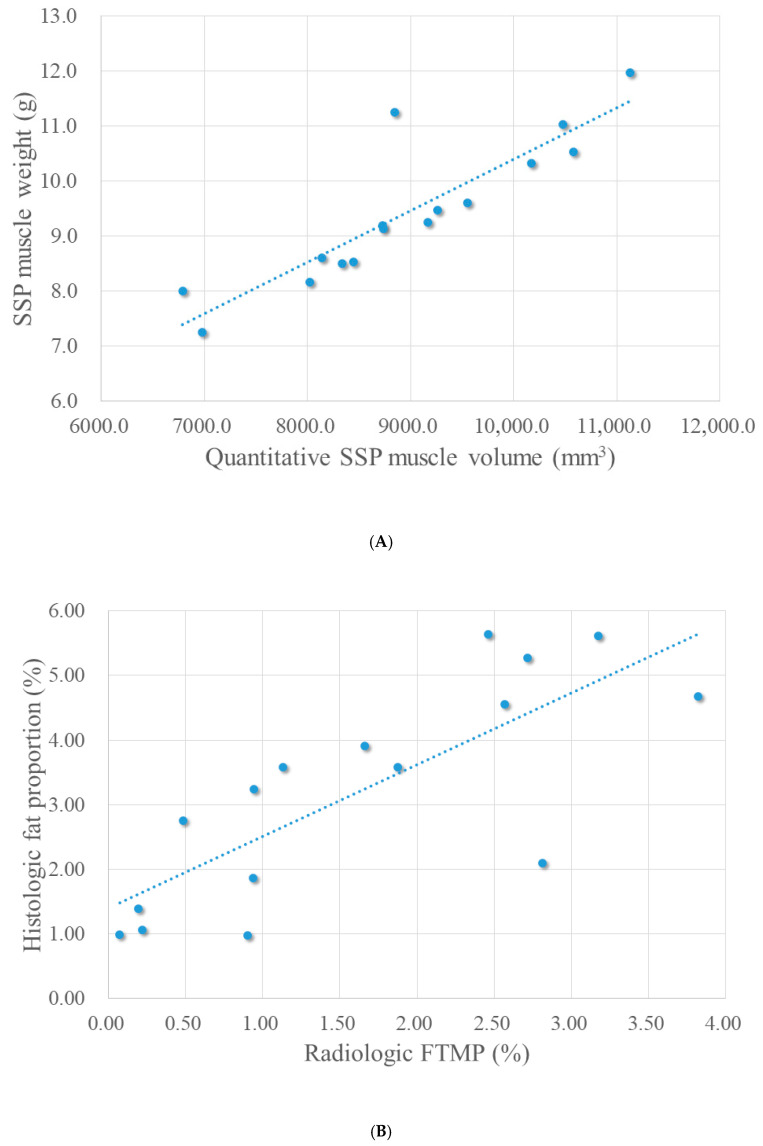
Scatter plots showing significant Spearman correlation between radiological and histological measurements. (**A**) Positive correlation between the supraspinatus muscle weight and the quantitative supraspinatus muscle volume (r = 0.887, *p* < 0.001). (**B**) Positive correlation between the histologic fat proportion and radiologic fat-to-muscle proportion (r = 0.784, *p* < 0.001). SSP, supraspinatus; FTMP, fat-to-muscle proportion.

We evaluated the statistical correlation between the supraspinatus muscle weight and quantitative supraspinatus muscle volume calculated from the 3D-reconstructed MRI data. Additionally, the correlation between the radiologic FTMP and the histologic fat proportion was analyzed. Spearman’s correlation analysis revealed strong and statistically significant positive correlations between the supraspinatus weight and quantitative muscle volume (r = 0.887, *p* < 0.001), as well as between the radiologic FTMP and histologic fat proportion (r = 0.784, *p* < 0.001) (Figure 7).

### 3.5. Biomechanical Evaluation

**Figure 8:**
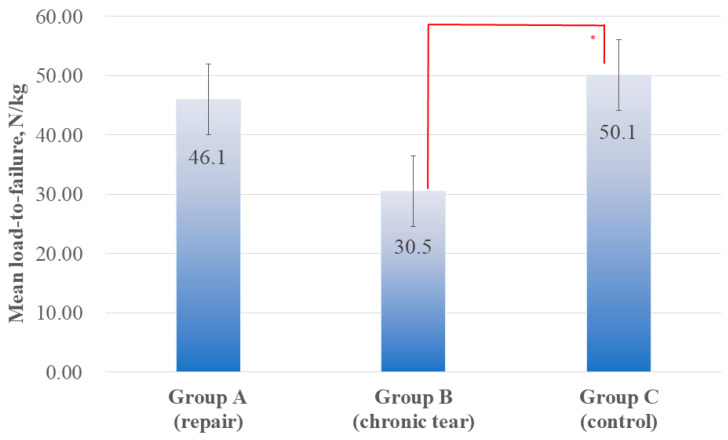
Comparison of ultimate load-to-failure among the three groups. The load-to-failure was significantly different among the groups (*p* = 0.027). Group C exhibited a significantly higher failure load compared with group B (*p* = 0.034), while no significant difference was observed in groups A and C (*p* = 1.000). The red asterisks indicate statistical significance based on the Bonferroni post hoc test (*p* < 0.05).

Ultimate load-to-failure was significantly different among three groups (*p* = 0.027). In post hoc analysis, group C presented a higher load-to-failure value (50.1 ± 4.1 N/kg) than group B (30.5 ± 3.8 N/kg, *p* = 0.034), while no significant difference was observed between group A (46.1 ± 19.5 N/kg) and group C (*p* = 1.000). Although group A showed a higher failure load compared with group B, the difference did not reach statistical significance (*p* = 0.109) (Figure 8).

Regarding the failure mode, midsubstance tears, which indicate a robust tendon-to-bone insertion, were most prevalent in group C (5/6, 83.3%). In contrast, group B showed the highest rate of bony avulsion (4/6, 66.7%), suggesting weakened structural integrity at the footprint. Group A demonstrated an intermediate recovery pattern, with midsubstance tears occurring in 66.7% (4/6) of the specimens. However, these differences in failure modes among the groups did not reach statistical significance (*p* = 0.195).

## 4. Discussion

In the present study, we evaluated a new quantified volumetric measurement for FI of RC muscles using MRI and 3D Slicer software in a chronic tear RCT rabbit model. The 3D-reconstructed muscle volume was highly consistent with the actual supraspinatus muscle weight (r = 0.887, *p* < 0.001), and the radiologic FTMP showed a strong correlation with histologically measured fat proportions (r = 0.784, *p* < 0.001). These findings indicate that our MRI-based quantitative measurement could represent the underlying degenerative changes in RC muscles following chronic tendon detachment. Moreover, the observed inverse relationship between radiologic FTMP and biomechanical strength suggests that this quantitative imaging parameter also reflects functional deterioration of the integrity of the bone–tendon interface. Therefore, these findings support the utility of 3D MRI-based volumetric analysis as a non-invasive surrogate for structural muscle quality and biomechanical integrity in chronic RCTs.

The clinical importance of FI of RC muscles lies in its substantial impact on the postoperative outcomes following RCR [6,20,21]. Severe FI is a well-established predictor of poor tendon reparability and is closely associated with increased healing failure rates [22,23]. Consequently, accurately evaluating FI is essential for predicting surgical outcomes, planning postoperative rehabilitation, and managing patient expectations.

Although the Goutallier–Fuchs grading system remains the clinical standard for assessing FI, its reliance on subjective visual assessment has resulted in suboptimal reliability across numerous studies [4,11,24]. Previous studies have shown that the interobserver reliability for Goutallier grading often falls within a fair to moderate range, with kappa values as low as 0.10 on MRI [24] and intraclass correlation coefficients (ICCs) ranging from 0.43 to 0.63 on CT arthrography [11]. Furthermore, Oh et al. [11] demonstrated that even among experienced radiologists and orthopedic surgeons, interobserver agreement remained only moderate on MRI (ICC: 0.60–0.72), and further decreased on CT (ICC: 0.43–0.60). This inconsistency is primarily attributed to the inherent subjectivity of semi-quantitative grading, in which assessments differ according to the clinical experience of observers.

Moreover, the conventional Goutallier–Fuchs grading system is inherently limited by its reliance on a single 2D sagittal oblique slice, the scapular Y-view [12]. Interpretation of a 3D structure using 2D imaging inevitably results in information loss and distortion. Considering that FI and muscle atrophy are heterogeneously distributed throughout the muscle, the limitations of 2D assessment are further amplified [25].

To address these limitations, various quantitative methodologies have been explored to assess the FI of RC muscles. These approaches can be categorized into two primary approaches, each contributing valuable objective data to the field. First, advanced imaging techniques such as Dixon-based MRI and MR spectroscopy (MRS) have been employed to provide precise measurements of intramuscular fat based on the distinct magnetic properties of fat and water protons [13,26,27,28,29,30]. A recent systematic review by Nasr et al. [30] demonstrated that multi-point Dixon MRI provided reliable and reproducible quantification of RC FI across diverse study populations, with inter-rater reliability generally ranging from moderate to excellent. However the significant heterogeneity in sequencing parameters and post-processing techniques remains a limitation. Furthermore, because MRS typically relies on single-voxel acquisition, it may not sufficiently capture the pathological condition of the entire muscle belly [26]. Second, 2D quantitative approaches based on signal intensity or CT attenuation have been introduced to enhance objectivity while utilizing conventional imaging protocols [31,32,33]. Xie et al. [33] proposed a signal intensity-derived fat percentage measurement from standard T1-weighted MRI using region-of-interest (ROI) analysis and identified improved diagnostic performance compared with the semi-quantitative Goutallier–Fuchs grading system. These methods showed strong correlations with Goutallier grades and demonstrated higher reliability than visual staging alone. However, because they remained inherently slice-dependent and were derived from limited 2D ROIs, they might not fully capture the heterogeneous 3D distribution of FI through the entire muscle volume. Additionally, signal intensity-based measurements can be influenced by variations in water content and technical factors inherent to MRI acquisition, including partial volume effects and magnetic field inhomogeneity. While these limitations apply broadly to MRI-based quantification, slice-dependent and ROI-based approaches may be particularly susceptible to sampling bias when FI is heterogeneously distributed. Deraedt et al. [25] demonstrated that while quantitative 2D methods possessed high reproducibility, they tended to overestimate the actual FI percentage by 4% to 6% compared to 3D measurements for Goutallier grades 2 to 4. This discrepancy often arises because fat is non-uniformly distributed throughout the muscle, and a single 2D slice can be altered by muscle retraction or structural shifts following surgical repair, potentially leading to inconsistent longitudinal assessments.

The present study builds upon these quantitative foundations by employing 3D reconstructed volumetric analysis to evaluate the RC muscles in their entirety. Recently, Yang et al. [34] demonstrated the validity of T1 mapping in monitoring FI progression in a rat model, sharing our framework of using MRI as an objective surrogate validated through direct biochemical correlation. However, while Yang et al. employed T1 mapping to provide a quantitative signal-based assessment of FI progression, our approach emphasizes volumetric quantification through 3D reconstruction. Unlike signal-based T1 mapping, our 3D Slicer-based method provides direct structural data on the entire muscle volume, effectively capturing the heterogeneous distribution of FI. By integrating the entire muscle volume, our methodology minimizes the potential sampling errors and provides a more comprehensive structural perspective on RC muscle degeneration. Consequently, our approach offers a quantitative and objective tool to complement both conventional grading and equipment-dependent imaging technologies. Importantly, our approach integrates whole-muscle volumetric assessment with direct histologic and biomechanical validation, a combination that remains uncommon in the current literature.

Beyond the technical advantages of 3D volumetric analysis, the use of an animal model in this study provided a unique opportunity to overcome the inherent limitations of clinical human studies. In human clinical research, it is often challenging to account for confounding variables such as age, gender, underlying comorbidities, and, most importantly, the exact chronicity of the RCT. By utilizing a standardized animal model, we were able to strictly control these variables, ensuring that the observed changes in FI and muscle volume were directly attributed to the induced tendon pathology. Furthermore, this experimental design allowed for a direct validation that is rarely possible in human subjects. Specifically, we were able to correlate 3D-reconstructed MRI data with the actual physical weight of the harvested muscles and quantitative histological fat proportion. This direct comparison confirms that our quantitative measurements accurately reflect the true physical and pathological state of the degenerated muscles. Crucially, the pathological progression of muscle degeneration following an RCT in this animal model closely mimics the characteristics of chronic human tears, including muscle atrophy and accumulation of intramuscular fat. By demonstrating the high accuracy and reliability of our 3D volumetric method in a precisely controlled pathological model, we have established a robust foundation for its clinical translation.

Despite the significant findings, this study has several limitations that should be addressed. First, the threshold values used to distinguish muscle from fat tissue may vary depending on institutional settings, including magnetic field strength, imaging protocol, and manufacturer-specific algorithms [14]. The signal intensity profiles and contrast-to-noise ratios at our 7.0T high-field strength differ significantly from those of standard clinical 1.5T or 3.0T MRI systems. Consequently, the specific threshold of 3600 identified in this model cannot be directly applied to clinical scanners. However, we have provided a detailed, reproducible framework that clinicians can adapt to establish protocol-specific thresholds for any MRI machine. Second, while a strong correlation exists between the MRI-derived FTMP and histological fat proportion, a disparity was observed in their absolute values. For instance, in Group B, the mean radiological FTMP (2.8%) was lower than the histological fat proportion (5.2%), suggesting that the MRI-based quantitative method may systematically underestimate absolute fat content due to the partial volume effect, where multiple tissue types within a single voxel result in averaged signal intensities. Therefore, this radiological parameter should be interpreted as a reliable tool for tracking longitudinal trends and providing objective comparisons rather than being considered equivalent to direct histological measurements. Third, while 3D Slicer is a validated open-source tool for medical image analysis, its application in providing volumetric data for FI of RC muscles is relatively novel. Additionally, although histology is the “gold standard” for validation, it is inherently a 2D methodology. To mitigate this, we analyzed multiple representative sections per specimen. However, 2D sections may still not fully represent the complex 3D heterogeneity of FI through the entire muscle belly. Fourth, although we measured the volume and fat fraction of the muscles, the manual segmentation process remains time-consuming, requiring approximately 30 min per case. While the assessment time decreased as the researcher became accustomed to the software, it remains a barrier to immediate clinical application. However, as 3D Slicer is an open-source platform, we anticipate the development of automated modules or machine-learning algorithms in the near future that can simultaneously segment multiple tissue ranges, thereby significantly enhancing time efficiency. Fifth, the sample size in this study was relatively small. However, to ensure statistical validity, we performed pre-experimental power analysis to determine the minimum number of subjects required to detect significant differences. While two subjects had to be excluded during the imaging processing phase due to significant MRI artifacts, the final number of included specimens still satisfied the requirements established by the power analysis. This ensures that the study maintains sufficient statistical power to support our findings. Furthermore, by utilizing a precisely controlled animal model, we minimized the biological heterogeneity often found in human cohorts, which allowed for a more reliable assessment even with a focused sample size. Finally, while our study established the validity of the 3D approach, it is important to acknowledge that it did not involve a direct head-to-head comparison with the Goutallier–Fuchs grading system or interobserver reliability assessments of 2D methods. Therefore, we emphasize the high accuracy of our method as a validated experimental framework, rather than claiming direct clinical superiority over existing 2D grading systems without comparative data. Subsequent clinical trials involving human patients using standard clinical MRI are necessary to confirm its diagnostic utility and comparative strength in clinical practice.

We believe our current findings in a validated pathological model provide a strong scientific foundation for these future human studies, ultimately aiming to improve diagnostic accuracy and surgical outcomes in clinical practice.

## 5. Conclusions

Direct comparison with histologic measurement validates the reliability of 3D MRI-based quantitative volumetric assessment for evaluating FI of RC muscles in a chronic RCT rabbit model. This approach may address the inherent limitations of the Goutallier–Fuchs grading system.

## Figures and Tables

**Table 1:** Supraspinatus muscle weight and muscle-to-body weight proportion ^a^.

	A (Repair)	B (Chronic Tear)	C (Control)	*p* Value
SSP muscle weight (g)	9.4 ± 1.2	8.5 ± 0.8	10.4 ± 1.1 ^b^	0.028
SSP muscle-to-body weight proportion (%)	0.23 ± 0.02	0.22 ± 0.02	0.25 ± 0.01 ^b^	0.031

^a^ Results are expressed as a mean ± standard deviation. *p*-values were determined using one-way analysis variance (ANOVA) followed by Bonferroni post hoc analysis for multiple comparisons. SSP, supraspinatus. ^b^ Statistically significant difference (*p* < 0.05) compared with group B.

**Table 2:** Quantitative MRI volumetric data of the supraspinatus muscle ^d^.

	A (Repair)	B (Chronic Tear)	C (Control)	*p* Value
Total volume (mm^3^)	8680.0 ± 548.7	8340.0 ± 1257.2	10,091.5 ± 920.8 ^b^	0.020 ^e^
Muscle volume (mm^3^)	8520.7 ± 521.9	8101.4 ± 1183.4	10,043.8 ± 911.0 ^a,b^	0.009 ^e^
Fat volume (mm^3^)	159.3 ± 74.7	238.6 ± 92.8 ^c^	47.6 ± 38.6	0.002 ^e^
FTMP	1.8 ± 0.8 ^c^	2.8 ± 0.7 ^a,c^	0.5 ± 0.4	<0.001 ^e^

^a^ Statistically significant difference (*p* < 0.05) compared with group A (repair group). ^b^ Statistically significant difference (*p* < 0.05) compared with group B (chronic tear group). ^c^ Statistically significant difference (*p* < 0.05) compared with group C (control group). ^d^ Results are expressed as a mean ± standard deviation. *p*-values were determined using one-way analysis variance (ANOVA) followed by Bonferroni post hoc analysis for multiple comparisons. FTMP, fat-to-muscle proportion. ^e^ Statistically significant difference.

**Table 3:** Quantitative histologic fat proportion using ImageJ ^a^.

	A (Repair)	B (Chronic Tear)	C (Control)	*p* Value
Fat proportion (%)	3.0 ± 1.2	5.2 ± 0.9 ^c^	1.7 ± 1.0	<0.001 ^b^

^a^ Results are expressed as mean standard deviation. *p*-value was determined using one-way analysis of variance (ANOVA) followed by Bonferroni post hoc analysis for multiple comparisons. ^b^ Statistically significant difference. ^c^ Statistically significant difference (*p* < 0.05) compared with group C (control group).

## Data Availability

The original contributions presented in this study are included in the article. Further inquiries can be directed to the corresponding author.
